# Luxation tibio-astragalienne pure chez un jeune sportif

**DOI:** 10.11604/pamj.2016.23.17.8311

**Published:** 2016-01-26

**Authors:** Mohamed Amine Karabila, Mohamed Kharmaz

**Affiliations:** 1 Service de Chirurgie Orthopédique et de Traumatologie, CHU Ibn Sina, Rabat, Maroc

**Keywords:** Cheville, luxation, pure, Ankle, dislocated, pure

## Image en medicine

Nous rapportons le cas d'un sportif de 20 ans victime d'un accident de hand-ball occasionnant une luxation pure de la cheville (A). La réduction a été pratiquée en urgence sous anesthésie générale, la radiographie et le scanner de contrôle après réduction avaient objectivés une bonne congruence articulaire (B, C). Une contention par une botte plâtrée fut assurée pendant six semaines. L'examen de la cheville réalisé après l'ablation du plâtre n'a pas retrouvé une laxité de la cheville et l'IRM de la cheville réalisée à la recherche d'une lésion ligamentaire avait objectivée une intégrité des ligaments péri-articulaire (D). Après un recul de neuf mois, les résultats fonctionnels étaient excellents, sans signes d'instabilité ni d'arthrose. La luxation tibio-talienne sans fracture malléolaire associée est une lésion très rare, souvent causée par un traumatisme de haute vélocité, jusqu’à l'heure actuelle, peu de cas ont été rapportés dans la littérature. La rareté de cette lésion peut être expliquée par la durabilité des ligaments par rapport aux malléoles et donc lors d'un traumatisme de la cheville une fracture se produise plutôt qu'une luxation.

**Figure 1:**
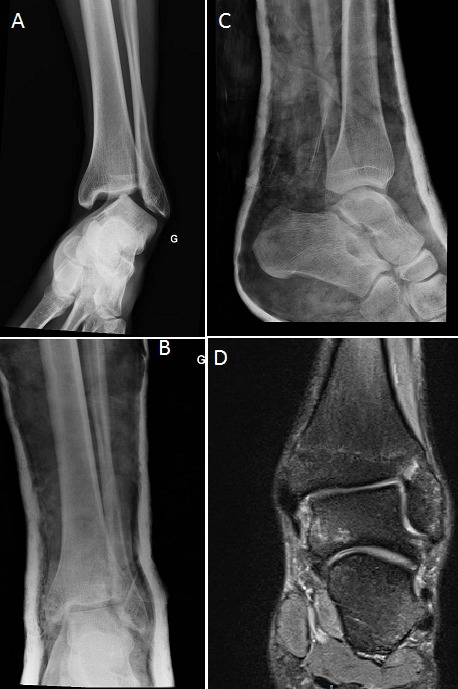
A) radiographie de la cheville gauche montrant la luxation; B) radiographie de la cheville de face après réduction; C) radiographie de la cheville de profil après réduction; D) IRM de la cheville de contrôle montrant intégrité des ligaments péri-articulaire

